# Multimorbidity, Treatment, and Determinants among Chronic Patients Attending Primary Health Facilities in Tshwane, South Africa

**DOI:** 10.3390/diseases11040129

**Published:** 2023-09-26

**Authors:** Thandiwe Wendy Mkhwanazi, Perpetua Modjadji, Kabelo Mokgalaboni, Sphiwe Madiba, Rifqah Abeeda Roomaney

**Affiliations:** 1 Department of Public Health, School of Health Care Sciences, Sefako Makgatho Health Sciences University, 1 Molotlegi Street, Ga-Rankuwa, Pretoria 0208, South Africa; 2 Non-Communicable Diseases Research Unit, South African Medical Research Council, Tygerberg, Cape Town 7505, South Africa; 3 Department of Life and Consumer Sciences, College of Agriculture and Environmental Sciences, University of South Africa, Florida Campus, Roodepoort 1709, South Africa; 4 Faculty of Health Sciences, University of Limpopo, Polokwane 0700, South Africa; 5 Burden of Disease Research Unit, South African Medical Research Council, Parowvallei, Tygerberg, Cape Town 7505, South Africa

**Keywords:** multimorbidity, medication adherence, determinants, out-patients, primary health facilities, South Africa

## Abstract

The growing burden of non-communicable diseases amidst the largest burden of HIV in South Africa leads to disease combinations of multimorbidity with the complexity of care. We conducted a cross-sectional study to assess multimorbidity, medication adherence, and associated factors among out-patients with chronic diseases in primary health care (PHC) facilities in Tshwane, South Africa. A structured questionnaire was used to collect data on comorbidities and medication adherence, along with socio-demographic and lifestyle factors. Logistic regression models were used to analyse the determinants of multimorbidity and medication adherence. In all 400 patients with chronic diseases (mean age: 47 ± 12 years) living in poor environments, common chronic conditions were hypertension (62%), diabetes (45%), HIV (44%), TB (33%), hypercholesterolemia (18%), and gout (13%). The proportion of concordant comorbidity (i.e., diseases with similar risk profiles and management) was 72%, more than 28% of discordant comorbidity (i.e., diseases not related in pathogenesis or management). Most patients had two coexisting chronic conditions (75%), while few had more than two chronic conditions (23%) and single-occurring conditions (2%). Prevalence rates for common multimorbidity patterns were 25% (HIV and TB), 17% (hypertension and diabetes), 9% (hypertension, diabetes, and hypercholesterolemia), and 2% (hypertension diabetes and HIV), while medication adherence was estimated at 74%. In multivariate analysis, multimorbidity was associated with an older age and lower socio-economic status, while medication non-adherence was associated with a younger age and socio-economic factors. The study highlights the presence of multimorbidity among primary care patients attributed to hypertension, diabetes, HIV, and TB in South Africa with non-adherence to medication in one-third of patients. Policies are needed for education on multimorbidity with a need to optimize lifestyle modifications, perhaps proactive outreach or nursing contact with high-risk patients with public-health-sensitive conditions, such as HIV and/or TB, as well as patients with a history of non-adherence to medications. Considerations should be given to the development of a medication adherence scale for multiple chronic conditions beyond assessing adherence to a single index medication.

## 1. Background

The concept of multimorbidity has become a priority agenda in various countries due to its dramatic increase and the strain put on health systems to manage patients with multiple chronic diseases [1]. Multimorbidity is the presence of two or more chronic diseases coexisting in an individual [2], affecting approximately 37% of adults, globally [3]. The prevalence rates of multimorbidity vary by socioeconomic status and regions [3], and for quite some time, high-income countries (HICs) were more affected (37%), with alarming rates of non-communicable diseases (NCDs). However, current literature reports an increase of multimorbidity (32.1%) in low-and middle-income countries (LMICs) [3,4], burdened by coexisting chronic diseases due to the aging of the population, but not exclusively affecting the elderly [5], while medication adherence remains low [6,7,8,9].

The absence of integrated chronic care models for the management of multiple coexisting burdens of NCDs and communicable diseases (CDs) among chronic patients in LMICs has been documented. These circumstances have been implicated in the ineffective management of multimorbidity in this region [10,11]. In particular, medication adherence (i.e., commitment to taking the treatment as prescribed) among patients with multimorbidity, which is a determining factor to maintain a longer healthy life, has raised concerns on poor management [8,12,13,14]. Over the past two decades, studies have reported a pill burden/polypharmacy of an average of five medications among multimorbid patients predisposing them to non-adherence [15,16]. Non-adherence to medication gives rise to poor clinical outcomes leading to a poor quality life, high mortality rates, and hospitalization, among others [17,18,19,20,21].

South Africa is currently experiencing a convergence of NCDs and CDs [22,23,24,25,26], causing the burden of multimorbidity to range from 3% to 87% in various group populations. The common multimorbidity patterns in South Africa are combined TB and HIV, hypertension and diabetes, and hypertension and HIV [27], and to a lesser extent, the coexistence of HIV, anaemia hypertension, and/or diabetes has been reported [28]. Although South Africa has put in place a primary health care approach for a better health care since the inception of the democratic government in 1994 [29,30], the country remains burdened with NCDs and CDs coexisting [13,23,25]. Despite the increasing co-occurrence of multiple chronic conditions and poor clinical outcomes among chronic patients [13], minimal research has been conducted on multimorbidity in South Africa [27,28]. While multimorbidity requires a complexity of care [13,31,32], most local studies have reported medication adherence from a single index point of view for hypertension, diabetes, and HIV treatments. The era of an anti-retroviral therapy (ART) programme in South Africa has improved the health of people living with HIV (PLWH) but has come along with cardiometabolic diseases [26,33,34].

The absence of integrated care in South Africa will increase the burden of multimorbidity and worsen the already burdened health systems and continues to affect the quality of individuals’ lives, especially multimorbid patients [23,26]. This calls for prioritized policies on the integrated management of NCDs and CDs [35], especially in a country like South Africa, which has an uneven distribution of healthcare needs from the public and private services, affecting mostly the socio-economically disadvantaged [36]. Therefore, considerations of a context-bound understanding of multimorbidity and treatment for the quality care of chronic patients are urgent [37]. In view of this, we assessed multimorbidity, medication adherence, and associated factors among out-patients with chronic diseases in primary health care (PHC) facilities in Tshwane, South Africa. Diagnosis, awareness and education, lifestyle modification, prescribed medication, and adherence/compliance, for successful clinical management, are pillars in the South African National user guide for the prevention and treatment of chronic diseases [38].

## 2. Methods

### 2.1. Study Design and Setting

A cross-sectional study was conducted between January 2022 and September 2022 guided by the Strengthening the Reporting of Observational Studies in Epidemiology (STROBE) [39]. In this study, we used a modified framework on multimorbidity and comorbid non-communicable and infectious chronic diseases (CNCICD) [40], adapted from the Innovative Care for Chronic Conditions (ICCC) framework of the World Health Organization (WHO) [41], focusing on the micro-level, which revolves around patients and the interaction with health facilities [40]. Together with CNCICD, the conceptual model for contributing factors to medication adherence, such as the patient, disease, treatment, healthcare system, and socioeconomic-related factors [42], informed data collection and analysis.

This study was conducted in the City of Tshwane municipality situated in the northern Gauteng Province of South Africa with 22 public PHC facilities [43,44]. Anecdotal information estimated approximately 269,700 patients from the respective facilities in 2019/2020. According to Statistics South Africa [45], a large proportion of the population is Africans (75.4%), followed by White (20.1%), then Coloured (2.0%), and Indian or Asian (1.8%) living across the informal settlements, rural, peri-urban, or urban settings of the City of Tshwane.

### 2.2. Sample Size, Sampling, and Participants

**Figure 1:**
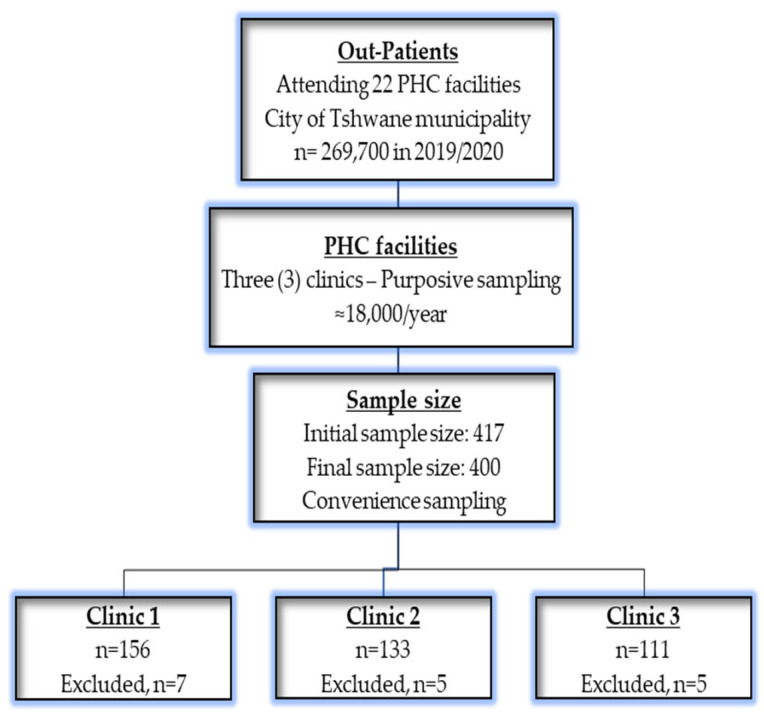
A flow chart of recruitment, sampling processes, and exclusions.

Out-patients with chronic conditions were estimated at approximately 4500 patients in each quarter, per year totalling 18,000 in a year. Considering a confidence level of 95%, 5% margin of error, and 50% response distribution, a minimum representative sample of 377 was calculated using Cochran’s formula using a validated Raosoft sample size calculator [46]. A sample of 377 was buffered with 10% to cater for non-response to increase the sample size to 415 out-patients. Out of the recruited 450 out-patients seeking chronic care at the selected facilities, 417 responded. Upon data capturing, 17 questionnaires had missing data above 10%, mainly on chronic conditions, and were excluded; seven patients were from clinic 1, five from clinic 2, and five from clinic 3, as indicated in Figure 1, below. A final sample size of 400 out-patients seeking chronic care at the selected facilities was eventually obtained.

First, the three facilities (i.e., Atteridgeville Clinic, Laudium Community Health Centre, and Lyttelton Clinic) where chronic health care services take place from Monday to Friday of every week were purposively selected out of the 22 facilities in the City of Tshwane municipality based on the highest population head count. Second, within the selected facilities, convenience sampling, applicable in clinical research [47], was used to select participants following the difficulty to obtain a random sampling due to long queues in the facilities and due to impatience of the out-patients waiting for hours. These patients were attending chronic care services in these PHC facilities, on treatment for the past 12 months at the times of the study, aged 18 years and above, and they gave informed written consent to participate in this study. A flow chart for recruitment, sampling processes, and exclusions is presented in Figure 1.

### 2.3. Data Collection and Tools

Following ethical approval by the Sefako Makgatho Health Sciences University Research and Ethics Committee (SMUREC/H/256/2021:PG) and permission from the Tshwane Research Committee in Gauteng Province, South Africa (GP_202112_005), the recruitment of patients started at one facility identified to conduct a pilot study prior to conducting the main study. From each of the three facilities selected, a healthcare worker was assigned to assist with the recruitment of chronic patients while in a queue for consultation in the morning. During recruitment, information leaflets written in three local languages, which are English, Setswana, and isiZulu, were distributed to the patients to explain the objectives of the study while in the chronic waiting area. The information leaflets had the contact numbers of the researcher provided for patients to enquire about the study, if need be, while other patients were able to seek clarity during the time of recruitment and consent to participate after consultation.

Through experts’ judgement, validity based on the content, construct, and face were performed to ensure the suitability of the questionnaire. Research assistants who were eloquent in English, Setswana, and isiZulu were trained to conduct preliminary interviews in a mini pilot study conducted in the smallest facility among 30 out-patients. The pilot study was purposed to assess the feasibility of the planned main study, especially based on recruitment processes, and data collection from chronic out-patients. The results from the pilot study, which were excluded from the analysis of the main study, showed that the questionnaire had relevant measurable variables, with easily arranged questions and layout. Minimal modifications were made to the questionnaire following the piloting outcomes. Therefore, we concluded that the main study would be feasible to be conducted among chronic patients at the selected public PHC facilities and continued to collect data on a larger scale.

The research team was made up of the principal researcher and two tutored research assistants administered a pre-tested questionnaire in either English, Setswana, or isiZulu languages preferred by the patient. The questionnaire was modified from previous similar studies carried out among patients with chronic conditions and collected demographic information and access to treatment and adherence [4,48,49,50,51,52,53,54,55]. Additional medical data were extrapolated from the patients’ file, and any information that was missing in the records was collected through self-reporting from patients.

Socio-demographic information included in the questionnaire entailed sex (male and female), age categories (18–39 years, 40–59 years, and ≥60 years), race (African, White, Coloured, and Asian), place of residence (informal settlements, peri-urban, and urban), marital status (single, married, and divorced), education level (no education, primary, secondary, grade 12, and tertiary education) and education level (i.e., completed grade 12 and tertiary), and employment status (employed, unemployed, or pensioner). Household information included the household income ($266.85; $266.85–$533.80; $533.80–$800.40; >$800.40), household size (<5 and ≥5 members), type of house [shack, RDP (Reconstruction and Development Programme) house and brick], electricity (no or yes), use of a refrigerator (no or yes), water access (no or yes), electricity access (no or yes), and type of a toilet (pit latrine or flush toilet), adapted from other studies conducted on chronic diseases [50,51].

Using WHO standard procedures and classifications [56], weight (using the smart D-quip electronic scale), height (using stadiometer/measuring board), and waist and hip (using a non-stretchable plastic tape measure) were measured three times to the nearest 0.1 from the participants. The calculated body mass index (BMI) defined underweight (<19 kg/m^2^), normal (19–24.99 kg/m^2^), overweight (≥25 kg/m^2^), and obesity, (≥30 kg/m^2^), while abdominal obesity was defined as waist circumference (WC) ≥88 cm, waist–hip ratio (WHR) ≥0.85, and waist-to-height ratio (WHtR) ≥0.5.

Multimorbidity was determined using a self-administered Co-Questionnaire (SCQ) enlisting medical conditions, such as diabetes, hypertension, heart, kidney, and lung diseases, as well as blood diseases, anaemia, etc. [55]. Participants were asked about the duration of the chronic diseases and the chronic condition that was diagnosed first. In addition to single conditions, patterns of multimorbidity were categorized as two conditions, three conditions, and four conditions of chronic diseases coexisting. Comorbidity classes were categorized as concordant, diseases with a similar risk profile and management (e.g., diabetes and hypertension), whereas discordant was described as diseases not related in pathogenesis or management (e.g., hypertension and HIV) [4,52] and were categorized by the research team based on their expertise.

Medication adherence was estimated using a General Medication Adherence Scale (GMAS) based on questions about treatment and the appointment and scored 0 to 20 in a three-level Likert score of zero to two points. GMAS ≥ 16 (≥80%) was labelled as adherence to medication, while non-adherence was GMAS < 15 (<80%) [48,53,54].

### 2.4. Data Analysis

STATA version 18 (StataCorp. 2018. Stata Statistical Software: Release 18. College Station, TX, USA) was used to analyse the data. Descriptive statistics (frequencies (n) and proportions (%)) were computed for the demographic, anthropometric, medication adherence, and morbidity characteristics of patients and compared by sex and age using a Chi square (χ^2^) test. Multimorbidity classes and medication adherence were considered as the two dependent variables, and binary logistic regressions were used to identify their determinants. The covariates were socio-demographic variables, anthropometric measurements, and selected behavioural and biological risk factors. Variables that were found to have an association with dependent variables at *p*-value < 0.25 in the binary logistic regression were entered into a multivariable logistic regression model. The magnitude of the association between independent and dependent variables was measured using odds ratios and the 95% confidence interval (CI) with the significant level (*p*-value < 0.05).

### 2.5. Ethics Committee

The Research and Ethics Committee, Sefako Makgatho Health Sciences University (SMUREC) approved the study (SMUREC/H/256/2021:PG). Further permission was obtained from Tshwane Research Committee in Gauteng Province, South Africa (GP_202112_005). Patients gave informed written consent for their participation, and the study adhered to ethical principles laid down in the Declaration of Helsinki [57].

## 3. Results

### 3.1. Characteristics of Patients

In all, 400 out-patients seeking chronic care at the Tshwane PHC facilities participated in the study. Their mean age was 47 ± 12 years, ranging from 25 to 73 years. Age groups were categorized into younger patients [<35 years, n = 70 (18%)], middle-aged patients [35–59 years, n = 236 (59%)], and older patients [≥60 years, n = 94 (23%)]. Two hundred and thirty-three patients were females [n = 233 (58%)], and one hundred and sixty-seven [n = 167 (42%)] were males. Most patients were Africans [n = 247 (62%)], while 14% (n = 56) were White, 12% (n = 48) were Coloured (i.e., person of mixed White, with either African or Asian ancestry), and 12% (n = 49) were Indians/Asians, residing across various settings, but mostly in the urban setting [n = 162; (41%)]. Being single (43%), attained secondary education (43%), and unemployed (40%) were prevalent characteristics in the sample population. Almost half of the participants came from households with a monthly income of $266.85–$533.80 per month. General overweight (23%), obesity (35%) by weight and height, and abdominal obesity by waist circumference (88%) were observed among the patients, in addition to irregular exercise (64%), salt intake (53%), current alcohol use (72%), and current cigarette smoking (53%) (Table 1).

### 3.2. Characteristics of Comorbidity

In Table 2, hypertension was the self-reported first diagnosed (49%) chronic condition and the most common condition (62%), while HIV (37%) was the second diagnosed, followed by diabetes as the third (10%) and the second (45%) prevalent chronic condition. HIV (44%) and TB (33%) were observed among patients. Other reported chronic conditions were hypercholesterolemia (18%), gout (13%), lung diseases (5%), anaemia (0.25%), and prostate cancer (0.25%). Three and more therapies (42%) were common for hypertension and the mostly used medications were Amlodipine besylate and Hydrochlorothiazide. For diabetes, monotherapy was prevalent, and Metformin and Glyburide were commonly used medications. Almost all patients were on ART (99%) for HIV treatment, and RH, Cotrimaxole, and Pyrodixine were used to treat TB. The hospitalization of chronic patients was higher among those with hypertension (17%), followed by diabetes (12%) and HIV (10%), and none with TB were hospitalized due to the condition. Using GMAS, low adherence had a score <15 (<80%) and high adherence had a score ≥16 (≥80%). The overall prevalence of medication adherence among out-patients was estimated at 74% (95%CI: 70–78).

Table 3 shows the prevalence and patterns of morbidity among out-patients with chronic diseases. Patients with single chronic conditions, two conditions, three conditions, and four conditions were classified, as in the study of Pati et al. [58]. Furthermore, we considered the concordant (i.e., diseases with a similar risk profile and management) and discordant (i.e., diseases not related in pathogenesis or management) comorbidities by the known pathophysiologic pathways [4,52]. Thirty-one patterns of morbidities occurring as either single chronic conditions [9 (2%)], two conditions [300 (75%)], three conditions [80 (20%)], or four conditions [11 (3%)] were observed among patients. Concordant multimorbidity [n = 281 (72%)] was more prevalent than the discordant multimorbidity [n = 110 (28%)]. There were 13 patterns of two chronic conditions with the top six being HIV and TB (25%), followed by hypertension and diabetes (17%), hypertension and gout (9%), hypertension and HIV (7%), hypertension and hypercholesterolemia (6%), and diabetes and HIV (3%). The top three chronic conditions of multimorbidity out of the identified 12 patterns were hypertension, diabetes, and hypercholesterolemia (9%), followed by hypertension, diabetes, and gout (3%), hypertension diabetes and HIV (2%), and hypertension, diabetes, and asthma (2%). Very few four-chronic-condition patterns (n = 3) were observed, with hypertension, diabetes, HIV, and hypercholesterolemia as the most occurring pattern.

**Figure 2:**
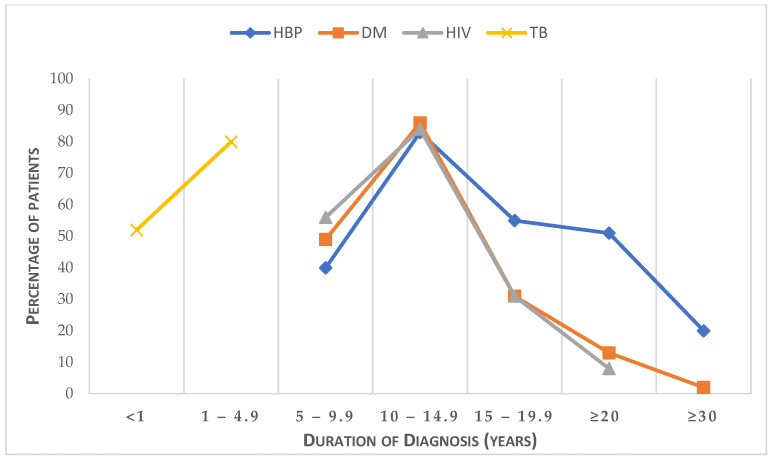
Self-reported duration of the diagnosis of HBP, DM, HIV, and TB among out-patients.

We assessed the duration of the self-reported diagnosis of HBP (i.e., hypertension), DM (i.e., diabetes mellitus), HIV, and TB among chronic out-patients, shown in Figure 2. It was observed that the diagnosis of TB was within one year prior to the study, while most patients with hypertension, diabetes, and HIV were diagnosed within the past 5 to 15 years, and the proportion of patients with chronic conditions decreased with the years of diagnosis, such as HIV (5%) towards 20 years and hypertension (28%) and diabetes (8%) for 20 years and above.

### 3.3. Factors Associated with Medication Adherence in Patients with Morbidity

In Table 4, chronic diseases among patients were compared by sex and age groups using a Chi-square test. There was an association between multimorbidity patterns with age (*p* ≤ 0.0001). Single chronic conditions were prevalent among the younger patients (9%) compared to the middle aged (1%) and older patients (0%), while three and four chronic conditions increased significantly with age. The association with multimorbidity was further observed in both men and women (*p* = 0.005), with no single chronic condition among men (0%) compared to women (4%), while the presence of two, three, and four conditions varied between men and women. Women (77%) exhibited better medication adherence than men (71%), although it was not significantly different (*p* = 0.164).

Table 5 shows the determinants of multimorbidity. First, univariate analysis (COR) showed associations of multimorbidity with age, marital status, education, employment, and household income (*p* < 0.25). After controlling for potential confounders (AOR), logistic regression showed multimorbidity associated with an older age [AOR = 14.95; 95%CI: 3.28–68.19] and higher income [AOR = 0.16; 95%CI: 0.03–0.71].

Table 6 shows the determinants of medication adherence. Univariate analysis (COR) showed an association of medication adherence with age, sex, residential area, marital status, education, employment, income, house type, household size, refrigerator use, current alcohol use, current cigarette smoking, BMI, WC, and WHtR (*p* < 0.25). After controlling for potential confounders, logistic regression showed that the odds of adherence to medication increased with age [≥60 years: AOR = 3.20; 95%CI: 1.03–9.94], marital status [cohabiting: AOR = 2.17; 95%CI: 1.02–4.63, married: AOR = 5.01; 95%CI: 1.97–12.69, and divorced: AOR = 8.84; 95%CI: 3.07–25.78], education [secondary: AOR = 3.06; 95%CI: 1.50–6.23, grade 12: AOR = 9.81; 95%CI: 3.35–28.77, and tertiary: AOR = 9.40; 95%CI: 3.39–26.82] and decreased with household size [AOR = 0.28; 95%CI: 0.15–0.55] and current cigarette smoking [AOR = 0.42; 95%CI: 0.23–0.76].

## 4. Discussion

This study assessed multimorbidity, medication adherence, and associated factors among out-patients with chronic diseases. The findings of this study showed the presence of multimorbidity patterns and classes among primary care out-patients in Tshwane, South Africa. The most common condition was hypertension (62%), which was self-reported as diagnosed first among almost half of the patients (49%), followed by diabetes (45%) as the second most prevalent chronic condition. High prevalence rates (21% to 77.9%) have been recorded in South Africa and remain the highest in the Southern Africa region [59,60], while the prevalence of diabetes has tripled since 2000 to date [61,62,63,64] with concerns of self-management [51,65]. The prevalence of hypertension and diabetes have been reported to increase significantly with age in South Africa [66,67] and in other countries, such as Kenya, [66] Ghana [66], Tanzania [68], and the United Arab Emirates (UAE) [48]. The current study also recorded a high occurrence of HIV as the condition that was diagnosed second among over one-third of the patients, and it was prevalent in younger patients, as reported previously [69]. South Africa has the highest HIV prevalence (18%) and supports the largest antiretroviral therapy (ART) [70]. The country is included in the WHO’s list of 30 high-burden tuberculosis countries and has one of the highest incidence rates of notified tuberculosis in the world with 55% attributed to HIV [71]. A growing burden of NCDs coexisting with CDs is currently leading to multimorbidity [23,24,72].

A poor socioeconomic status in terms of low household income per month, unemployment, and dependence on old age pension grants was observed in this study. Social inequalities have been associated with multimorbidity, suggesting that individuals who are socioeconomically deprived tend to have more chronic conditions than the least deprived ones [73,74,75]. This was corroborated in the current study showing that patients who came from households with high monthly income had lower odds of multimorbidity compared to those coming from households with low monthly income. Similarly, previous studies have reported an earlier onset of multimorbidity in patients living in the most deprived areas compared with the most affluent areas [10,76,77]. Therefore, addressing inequalities regarding healthcare use is critical to achieve better health outcomes for multimorbid patients living in poor backgrounds [78]. Furthermore, the current study showed a high prevalence of poor lifestyle factors, such as overweight/obesity, salt intake, irregular exercise, alcohol use, and smoking, which are implicated in the development and progression of chronic diseases [10]. These lifestyle factors have been associated with an increase in multimorbidity, in addition to population ageing [10,79]. In particular, alarming rates of overweight/obesity are being observed in South Africa, higher than in other Africa continues, and almost similar as in HIC countries [51,80,81].

The current study further showed that single chronic conditions (2%) were diminishing among the chronic patients, while concordant comorbidity (72%), mainly combined diabetes and hypertension, was common than the discordant class (28%). Concordant comorbidity was prevalent among older patients (≥60 years of age), while discordant multimorbidity was common among younger patients (≤35 years). Prior studies showed similar patterns of disease with hypertension and diabetes being the most common concordant comorbidity disease cluster [14,58,82]. Hypertension and diabetes are known to frequently co-occur and share common risk factors and complications that include microvascular and macrovascular disorders [83,84]. The prevalence of concordant comorbidity was higher compared to reports in South Africa (6%) [85] and Africa (55.8%) [4], but lower than in countries, like India (84%) [58], Spain (82%) [86], the Netherlands (84.6%) [87], and USA (88.5%) [88].

Regarding multimorbidity patterns, our study indicated that a large component of multimorbidity was sequentially attributed to hypertension, diabetes, HIV, and TB. Three quarters of the patients had two chronic conditions, one quarter had more than two conditions, and very few had single conditions. Coexisting HIV and TB was the common pattern of two chronic conditions. That was followed by hypertension and diabetes, hypertension and gout, and hypertension and HIV. The three-condition patterns of multimorbidity were hypertension, diabetes, and hypercholesterolemia, hypertension, diabetes, and gout, and hypertension, diabetes, and HIV. A systematic review on multimorbidity in South Africa has reported hypertension as the most frequent condition, and it is associated with other diseases [27]. Furthermore, most of the above-mentioned multimorbidity patterns have been reported in South Africa [27] and occur commonly among primary care patients in South Africa [13,14,51,82,89]. The occurrence of multimorbidity is further consistent with the findings from several countries, like Brazil [19], India [90] Ghana [91], and the UAE [48]. The prevalence of multimorbidity, as reported in previous studies in South Africa, ranged from 3% to 87% [27,82,89], attributed to the difference in the methodologies used, the target population with various chronic diseases, and the context of socio-demographic variations [4,92]. Our findings are similar to several studies that reported higher odds of having multimorbidity among older out-patients compared to the younger group [4,10]. Therefore, it was not a surprise that no chronic condition occurred on a singular level in older patients (≥60 years). However, it is worth noting that over half of the patients in this study were below 60 years of age, emphasizing that multimorbidity is not just a feature of ageing [10,93]. Therefore, diseases can coexist in the same individual for several reasons, including common risk factors or mechanisms and iatrogenic conditions [94].

We further observed adherence to medication estimated at 74%, almost similar to the prevalence reported among patients with multimorbidity in the United Arab Emirates (78%) [48]. The challenge remains that we are not able to compare our findings with other studies in South Africa and African countries, since most studies reported medication adherence to a single chronic illness. One-third of patients with multimorbidity in this study were not adhering to medication, supporting the notion of the complexity of medication adherence and the self-management of multimorbidity among patients. This is consistent with a recent south African study that reported poor diabetic self-management among diabetes patients with two and three coexisting chronic conditions [51]. Adherence to medication was associated with older age, marital status, higher education, lower socioeconomic status, and current cigarette smoking. Clearly, these results demonstrate the multifactorial determinants involved in medication adherence, as reported by other researchers [48]. Similar to one study conducted in the UAE [48], we found that the odds of adherence to medication increased with age, and patients aged 60 years old and above were three times more likely to adhere to medication than their younger counterparts.

Lastly, most of the chronic patients in South Africa use PHC facilities for primary care treatment accessed through the Centralised Chronic Medicine Dispensing and Distribution Programme (CCMDD), and adherence to the treatment of single conditions is suboptimal, except for HIV [13,65,95]. The World Health Organization (WHO) calls for universal health insurance and health service coverage in LMICs, particularly for vulnerable groups [96], as well as the United Nations (UN)’ sustainable development goals (SDG) 3 on Good Health and Well-Being to ensure healthy lives and promote well-being for all at all ages [97]. The fact remains that treatment plans in patients with multimorbidity are complex and multifactorial. Therefore, improving medication adherence requires a multilevel approach from patient to healthcare factors.

## 5. Limitations

There are some limitations to this study. First, the proposed sample size calculation used in this study is for a simple random sampling strategy. Therefore, the use of non-probability sampling might have introduced selection bias, which we mitigated by obtaining a larger sample size of the participants. Second, in self-reported surveys, patients may overestimate their adherence to medications due to social desirability bias. Nevertheless, this might have been mitigated by using well-trained research assistants and a robust validated questionnaire with identified options to questions. Third, the lifestyle factor assessment depended largely on self-report measures, which can also be subject to social desirability bias, and hence, the result must be treated with caution. Fourth, a causal relationship of medical adherence and its correlates cannot be ascertained, given the cross-sectional design method adopted in the study. Furthermore, assessing salt intake, alcohol intake, and physical activity using standardized methods, such as biochemical parameters of sodium, the Alcohol Use Disorders Identification Test (AUDIT), and the Global Physical Activity Questionnaire (GPAQ), respectively, could have added more objectivity. Additional qualitative methodology for future research will enrich and improve the robustness in understanding the multidimensional factors related to medication adherence. Lastly, the findings may not be generalizable to the general population because this study used hospital out-patients. Nonetheless, in addition to reporting concordant versus discordant comorbidities, and multimorbidity patterns among out-patients in PHC facilities in Tshwane, this study has showed factors associated with medication adherence among multimorbid patients. Although, there is no standard criterion available to assess medication adherence in patients receiving multiple treatments, rendering an appropriate measurement of multiple medication adherence a challenge, to our knowledge, our study is among the very few in South Africa that has attempted to assess medication adherence among multimorbid patients, rather than for one single chronic condition.

## 6. Conclusions

This study highlights the presence of multimorbidity among primary care patients in Tshwane facilities, South Africa. A total of thirty-one multimorbidity patterns were observed among patients of all ages from the younger, to middle-aged, and older patients. Our study indicated that a large component of multimorbidity was sequentially attributed to hypertension, diabetes, and HIV. Almost one-third of chronic patients were non-adherent to medication, with increased odds among patients who are younger and with marital status, education, household size, and current cigarette smoking. Policies are perhaps needed for education on multimorbidity with a need to optimize lifestyle modifications, in addition to proactive outreach or nursing contact with high-risk patients with public-health-sensitive conditions, such as HIV and/or TB, as well as patients with a history of non-adherence to medications. Considerations should be given to the development of a medication adherence scale for multiple chronic conditions beyond assessing adherence to a single index medication.

## Figures and Tables

**Table 1:** Characteristics of out-patients with chronic diseases.

Variables	Categories	Frequency (n)	Percentage (%)
Sex	Male	167	42
	Female	233	58
Age groups	18–34	86	30
	35–59	133	47
	≥60	183	23
Race	Africans	247	62
	White	56	14
	Coloureds	48	12
	Asian	49	12
Place of Residence	Informal settlement	108	27
	Peri-urban (i.e., township)	133	33
	Urban	162	41
Marital status	Cohabiting	79	20
	Single	93	23
	Married	143	36
	Divorced	85	21
Level of Education	Primary	74	19
	Secondary	173	43
	Completed grade 12	55	14
	Tertiary	98	25
Employed	No	149	37
	Yes	160	40
	Pensioner	91	23
BMI (kg/m^2^)	Normal	8	2
	Underweight	160	40
	Overweight	92	23
	Obese	140	35
Waist circumference	Normal	49	12
	Abdominal obesity	351	88
Waist-to-hip ratio	Normal	239	60
	Abdominal obesity	161	40
Waist-to-height ratio	Normal	298	75
	Abdominal obesity	102	25
Household income/month	$266.85	54	14
	$266.85–$533.80	172	43
	$533.80–$800.40	73	18
	>$800.40	101	25
Regular exercise	No	257	64
	Yes	143	34
Salt intake	No	154	39
	Sometimes	36	9
	Yes	210	53
Current alcohol use	No	111	28
	Yes	289	72
current cigarette smoking	No	188	47
	Yes	212	53

**Table 2:** Characteristics of comorbidity among out-patients.

Variables	Categories	n (%)
First diagnosed condition *	Hypertension	197 (49)
	HIV	148 (37)
	Diabetes	38 (10)
	Lung disease (i.e., asthma/TB)	15 (4)
	Hypercholesterolemia	2 (1)
Hypertension	No	151 (38)
	Yes	249 (62)
Hypertension therapy	Mono	54 (22)
	Bi therapy	89 (36)
	≥3 therapies	106 (42)
Have blood pressure monitor	No	213 (86)
	Yes	36 (14)
Diabetes	No	219 (55)
	Yes	181 (45)
Diabetes therapy	Mono	148 (82)
	Bi therapy	30 (10)
	3 therapies	3 (2)
Have glucose meter	No	66 (36)
	Yes	116 (64)
HIV	Negative	223 (56)
	Positive	177 (44)
Taking ART (HIV)	No	1 (1)
	Yes	176 (99)
TB treatment	No	0(0)
	Yes	132 (100)
TB	Negative	268 (67)
	Positive	132 (33)
Other chronic conditions	None	243 (61)
	High cholesterol	77 (18)
	Gout	50 (13)
	Lung diseases,	18 (5)
	High cholesterol/gout	7 (1.75)
	High cholesterol/cardiac problems	3 (0.75)
	Anaemia	1 (0.25)
	Prostate cancer	1 (0.25)
Hospitalization due to:	Hypertension (yes)	249 (62)
	Diabetes (yes)	22 (12)
	HIV-related problems (yes)	18 (10)
	TB (yes)	0
Medication adherence **	Adherence	297 (74)
	Non-adherence	103 (26)

* Indicates that participants self-reported the condition that was diagnosed first; ** indicates self-reported medication adherence.

**Table 3:** Prevalence and patterns of morbidity among out-patients, and level of adherence.

Variables	Categories	n (%)
Multimorbidity classes	Concordant	281 (72)
	Discordant	110 (28)
Morbidity patterns	Single chronic conditions	9 (2)
	Two conditions	300 (75)
	Three conditions	80 (20)
	Four conditions	11 (3)
Two conditions	HIV and TB	98 (25)
	Hypertension and diabetes	67 (17)
	Hypertension and Gout	36 (9)
	Hypertension and HIV	28 (7)
	Hypertension and hypercholesterolemia	23 (6)
	Diabetes and HIV	13 (3)
Three conditions	Hypertension, diabetes, and hypercholesterolemia	36 (9)
	Hypertension, diabetes, and gout	10 (3)
	Hypertension, diabetes, and HIV	6 (2)
	Hypertension, diabetes, and asthma	6 (2)
Four conditions	Hypertension, diabetes, HIV, and hypercholesterolemia	7 (2)

Concordant refers to having diseases with a similar risk profile and management, and discordant refers to diseases not related in pathogenesis or management.

**Table 4:** Prevalence and patterns of morbidity among out-patients by sex and age group.

Variables	Males	Females	*p*	≤35	35–59	≥60	*p*
				Years	Years	Years	
Hypertension			0.061				≤0.0001
No	72 (43)	79 (34)		50 (71)	91 (29)	10 (11)	
Yes	95 (57)	154 (66)		20 (29)	145 (61)	84 (89)	
Diabetes			0.081				≤0.0001
No	100 (60)	119 (51)		61 (87)	124 (53)	34 (36)	
Yes	67 (40)	114 (49)		9 (13)	112 (47)	60 (64)	
HIV			0.213				≤0.0001
No	87 (52)	136 (58)		11 (16)	129 (55)	83 (88)	
Yes	80 (48)	97 (42)		59 (84)	107 (45)	11 (12)	
TB			≤0.0001				≤0.0001
No	94 (56)	174 (75)		26 (37)	156 (66)	86 (91)	
Yes	73 (44)	59 (25)		44 (63)	80 (54)	8 (9)	
Multimorbidity class			0.006				0.021
Concordant	132 (79)	149 (67)		45 (70)	158 (68)	78 (83)	
Discordant	35 (21)	75 (33)		19 (30)	75 (32)	16 (17)	
Morbidity patterns			0.005				≤0.0001
Single	0	9 (4)		6 (9)	3 (1)	0	
Two	131 (78)	169 (73)		58 (83)	183 (78)	59 (63)	
Three	28 (17)	52 (22)		6 (9)	44 (19)	30 (32)	
Four	8 (5)	3 (1)		0	6 (3)	5 (5)	
Medication			0.164				0.003
Adherence	118 (71)	179 (77)		43 (61)	174 (74)	0 (85)	
Non-adherence	49 (29)	54 (23)		27 (39)	65 (26)	14 (15)	

Concordant refers to having diseases with a similar risk profile and management, and discordant refers to diseases not related in pathogenesis or management.

**Table 5:** Association of multimorbidity with covariates among patients.

Multimorbidity	COR (95%CI)	*p*-Value	AOR (95%CI)	*p*-Value
Age (years)				
≤35	1		1	
>35	7.77 (1.90–31.75)	0.004	14.95 (3.28–68.19)	≤0.0001
Income ($)				
<$533.80	1		1	
≥533.80	0.38 (0.09–1.53)	0.172	0.16 (0.03–0.71)	0.017

**Table 6:** Determinants of medication adherence among patients.

Medication Adherence	COR (95%CI)	*p*-Value	AOR (95%CI)	*p*-Value
Age (years)				
<35	1			
35–59	1.88 (1.31–2.71)	0.001	2.15 (1.00–4.60)	0.051
≥60	1.53 (1.02–2.32)	0.042	3.20 (1.03–9.93)	0.049
Marital status				
Single	1			
Cohabiting	1.76 (0.95–3.27)	0.072	2.17 (1.02–4.63)	0.045
Married	3.78 (2.06–6.96)	≤0.0001	5.01 (1.97–12.69)	0.001
Divorced	8.48 (3.62–19.87)	≤0.0001	8.84 (3.07–25.78)	≤0.0001
Education				
No education/primary	1			
Secondary	2.34 (1.33–4.11)	0.003	3.06 (1.50–6.23)	0.002
Grade 12	5.37 (2.19–12.69)	≤0.0001	9.81 (3.35–28.77)	≤0.0001
Tertiary	6.43 (3.02–13.71)	≤0.0001	9.40 (3.39–26.82)	≤0.0001
Household size				
<5 members	1			
≥5 members	0.76 (0.48–1.19)	0.231	0.28 (0.15–0.55)	≤0.0001
Current cigarette smoking				
No	1			
Yes	0.30 (0.18–0.40)	≤0.0001	0.42 (0.31–0.76)	0.004

## Data Availability

The dataset for chronic patients generated and analysed during the current study are available from the corresponding author upon reasonable request.
